# How to deal with moral challenges around the decision-making competence in transgender adolescent care? Development of an ethics support tool

**DOI:** 10.1186/s12910-022-00837-1

**Published:** 2022-09-22

**Authors:** Janine de Snoo-Trimp, Annelou de Vries, Bert Molewijk, Irma Hein

**Affiliations:** 1 Department of Ethics, Law and Humanities, Amsterdam University Medical Centers, Location VUmc, Amsterdam, The Netherlands; 2 Department of Child and Adolescent Psychiatry, Center of Expertise on Gender Dysphoria, Amsterdam University Medical Centers, Location VUmc, Amsterdam, The Netherlands; 3 Center of Medical Ethics, Institute of Health and Society, University of Oslo, Oslo, Norway; 4 Department of Child and Adolescent Psychiatry, Amsterdam University Medical Centers, Location AMC, University of Amsterdam and Levvel, Amsterdam, The Netherlands

**Keywords:** Decision-making competence, Clinical ethics support, Ethics support tool, Transgender adolescents

## Abstract

**Background:**

Decision-making competence is a complex concept in the care for transgender and gender diverse adolescents, since this type of care concerns one’s developing gender identity and involves treatment options that often lack international consensus. Even despite competence assessments, moral challenges arise in the decision-making process. Here, traditional forms of clinical ethics support such as moral case deliberation might not fit as these do not provide thematic guidance. This study therefore aimed to develop a practice-oriented ethics support tool to assist care providers when dealing with moral challenges around decision-making competence in transgender adolescent care.

**Methods:**

The study followed a participatory design to develop a tool in close collaboration with care providers; they had a say in all phases of development and dissemination. Firstly, nine care providers were interviewed about experienced moral challenges and needs for ethics support. Based on this, the structure and content of the tool was constructed and discussed in two focus group meetings, after which four care providers tested the tool and additional feedback was collected from the team and an advisory board. The final tool was presented to all Dutch care providers in transgender adolescent care.

**Results:**

Care providers expressed a need for guidance in defining and assessing decision-making competence. Main moral challenges concerned discussing fertility options with young clients, dealing with co-occurring mental health difficulties and the decision-making role of parents. The final tool, named the Competence Consultant, is an interactive pdf containing four parts: (1) Clarify information; (2) Identify doubts and moral questions; (3) Guidance for conversations and (4) Overview and Conclusions.

**Discussion:**

Developing an ethics support tool in a controversial care setting is highly relevant as it aims to help individual care providers in defining, discussing and dealing with their moral challenges in actual practice. The ‘Competence Consultant’ for transgender care providers contributes to their moral sensitivity and moral competence. It is an example of the development of innovative and integrative forms of thematic ethics support.

**Supplementary Information:**

The online version contains supplementary material available at 10.1186/s12910-022-00837-1.

## Introduction

Informed consent and shared decision-making are core concepts in current healthcare practice, emphasizing both an open and full disclosure of information by physicians, as well as the active participation and involvement of patients to consent for and (co-)decide about their medical treatment [1, 2]. This participation requires a certain ability from patients: decision-making competence, which is generally assessed using four criteria: the ability to ‘communicate a choice’, ‘understand the relevant information’, ‘appreciate the situation and its consequences’ and ‘reason about treatment options’ [3]. Informed consent and hence the assessment of decision-making competence of patients, or “Gillick” competence in many countries in the Anglosphere, is needed before any intervention can be applied. It is different from the legal right to provide informed consent, which depends on age and differs across countries depending on their regulations [4]. In the care for transgender and gender diverse adolescents specifically, the assessment of decision-making competence is delicate and a special concern, because it concerns one’s gender identity, which inherently is self-determined while still in development at this stage, and involves decisions with potentially far reaching lifelong consequences, often made at a young age [5, 6]. Treatment decisions involve starting puberty suppression to pause the biological hormonal development, gender affirming hormonal treatment and (when reaching adulthood) gender affirming surgery [7]. According to clinical transgender care guidelines, one of the important criteria to start these medical interventions is that the adolescent has capacity to give informed consent [8, 9].

Decision-making in this setting involves many moral questions and ongoing international debates about children’s competence, minimum age, role of parents and the influence of social and societal contexts [6, 10–14]. Firstly, considering the earlier mentioned capacities for competence [3], these are yet limited and still developing in children and young adolescents [13]. Hence, questions can rise about what they are able to foresee and understand of possible treatment options and (long-term) consequences [15]. It is worried that they might not yet have developed a stable gender identity. Care providers might struggle with protecting the vulnerable adolescent for making uninformed or insufficiently considered decisions on the one hand and promoting their autonomy and possibilities for self-development on the other hand [13]. There is no clear international consensus about the age at which people can be deemed decision-making competent regarding this informed consent [13]. Transgender adolescents also themselves mentioned the moral struggle about defining the right age for being a decision-making partner for medical treatments [16].

Recently, the High Court in the UK ruled that minors are highly unlikely to be able to provide informed consent for puberty suppressing treatment [17]. It meant that decisions regarding puberty suppression had to be made with court involvement, causing extra barriers to the provision of medical affirming care for transgender adolescents. This court ruling was therefore considered as highly troublesome and harmful for this vulnerable group of adolescents suffering from an incongruence between their birth assigned sex and experienced gender and for the understanding of the concept of informed consent in general [18, 19]. Delaying puberty suppression treatment could not only deny existing suffering but may also result in more invasive treatment needs at a later age (such as a mastectomy). The ruling has meanwhile been overturned in higher court [20]. A recent empirical study which used the validated MacArthur Competence Assessment Tool for Treatment (MacCAT-T) to assess the competence of adolescents showed that the majority of adolescents was considered competent to consent to puberty suppressing treatment during regular informed consent procedures, elucidating that care providers most often do not doubt decision-making competence [21]. The current debate in international (empirical and societal) media nevertheless shows the complexities surrounding decision-making competence in young transgender people. To cite a recent editorial in the Lancet Child and Adolescent Health [22] on care for trans youth: *“…disproportionate emphasis is given to young people’s inability to provide medical consent”*, hence, the current lack of consensus about puberty suppression being an evidence-based form of treatment is often linked to the minor’s decision-making competence [23, 24].

However, despite this debate and beyond being deemed (in)capable of decision-making, several moral challenges still exist or arise in the decision-making process with transgender adolescents. They primarily and especially occur in the daily clinical practice of healthcare professionals. These moral challenges related to the decision-making competency of adolescents can and should be distinguished from uncertainties about the gender dysphoria itself and about access to transgender care. The same applies to challenges related to psychological discussions about how to reliably assess competence [25]. Moral challenges involve, for instance, concerns about the (in)stability of gender identity development and long-term impact on fertility [26] or dealing with co-occurring psychiatric problems such as autism [27]. Furthermore, care professionals might be confronted with moral questions around the role and responsibility of parents and their own professional responsibility in the decision-making process here [6, 12, 28].

The here mentioned moral issues all arise around assessing and dealing with the decision-making (in)competence of youth and their role in the decision-making process. These issues are often unsolvable at the moment that they occur, or even unsolvable at all, for instance because both transgender care providers as well as the adolescent and their parents might not be sure how future identity and treatment preferences will develop. This type of care inherently involves many uncertainties about the future, like long-term effects, or about gender identity development, that challenge determining an adolescent’s decision-making competence for informed consent. At the same time, there is stress and suffering of adolescents, and not much time to wait as puberty will (continue to) develop [18, 19]. Therefore, care providers, adolescents and other stakeholders should find ways to discuss and deal with the dilemmas even when ideal solutions cannot be found since the tragic character of a dilemma always involve some sort of (moral) harm [29].

In order to deal with moral challenges related to the decision-making competence of transgender adolescents and the decision-making roles, care providers might profit from structured and careful supporting procedures [6, 12, 30]. For this, various kinds of ethics support might be suitable. Several empirical studies on the evaluation of ethics support in health care in general [31, 32], and in transgender care specifically [33], showed positive results with respect to constructively dealing with moral challenges.

For example, a moral case deliberation session can be organized as a structured group conversation about moral questions and moral dilemmas with the guidance of a trained facilitator [34]. However, (planning) a moral case deliberation can be time-consuming and only offers a structure for general ethical reflection. In order to provide ethics support that can be used individually, flexible and that is tailored to the specific theme, ethics support tools have been developed [33, 35]. In this way, ethics support can be stronger integrated in the daily practice of transgender care professionals themselves [36, 37].

We started a practice-oriented study aiming to develop an ethics support tool, in order to assist care providers when dealing with moral challenges around decision-making competence in care for transgender and gender diverse adolescents. The tool should help to understand and weigh moral dilemmas around the decision-making competence when starting puberty-blocking treatment. Our aim was twofold: (1) to describe the moral dilemmas around assessing medical decision-making competence and the specific needs for ethics support according to care providers, and (2) to co-create a practical and specific hands-on ethics support tool to deal with these dilemmas and support the decision-making process in general.

## Methods

### Setting and context of the study

The research team consists of both care providers (AV and IH) from Child and Adolescent Psychiatry Department and the Amsterdam Center of Expertise on Gender Dysphoria (CEDG), as well as ethics support researchers from the department of Ethics, Law and Humanities at Amsterdam UMC (JS and BM). The ethics support team has a close connection with the CEDG as they have cooperated for more than a decade already [36]. The CEDG has a long history and important leading role, both nationally and internationally, in providing gender affirming treatment and conducting research on transgender care [38]. In the Netherlands, transgender care has been centralized in this center for a long time, while recently three new centers have started to provide (parts of) transgender care for (a lower number of) minors as well: in periphery centers in the northern (Zaandam) and southern (Genderteam Zuid) parts of the country, and in another academic hospital (RadboudUMC). Care providers from these new centers were involved in the current project as well.

The research was funded by the Janivo Stichting, a foundation for societal, research and/or cultural projects with children and adolescents.

### Co-creation

**Fig. 1:**
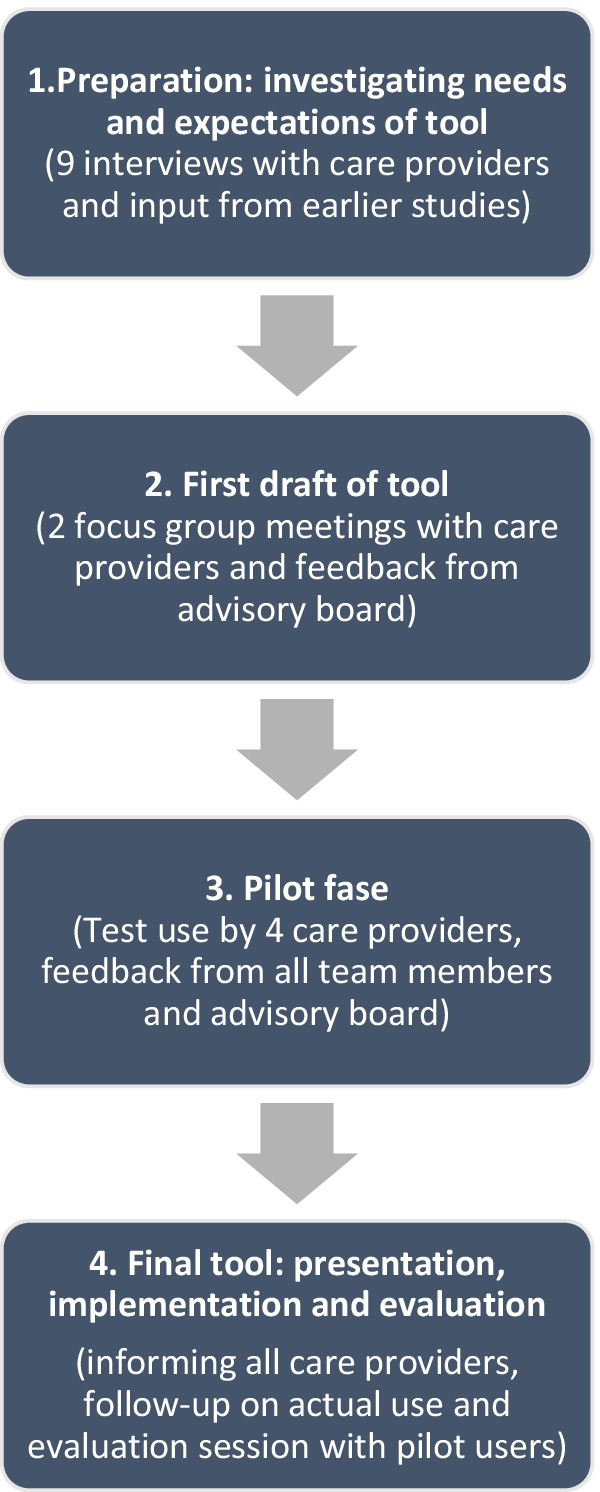
Process of co-creation

The study had a participatory design, as the tool was developed in close participation with the care providers for transgender and gender diverse youth. This co-creation or ‘collective making’ [39] was understood as ‘collaborative generation of knowledge by academics working alongside stakeholders’ [40], with an emphasis on the context, individual experiences, quality of relationships, creative innovative research and – because of this emphasis—believed to reach substantial impact [39, 40]. Input from care providers was collected via interviews and focus group meetings. A core working group of these care providers was formed to intensively discuss and develop the content, layout and use of the tool. We also integrated findings from six previous studies on both care providers’ and transgender adolescents’ views on their decision-making competence when making decisions for puberty blocking treatment [16, 21, 37, 41, 42] and moral dilemmas as experienced by several care teams across Europe [14] in our data analysis. Besides, an advisory board was formed with experts in child decision-making competence, medical ethics and transgender youth care, for periodical meetings for advice and feedback. The process of co-creation is presented in Fig. 1.

### Phase 1: interviews

Firstly, JS interviewed care providers from various disciplines and transgender care clinics in the Netherlands about their needs for and expectations of a tool (see Table 1). These professionals were purposively recruited to represent all relevant professions and collect views from both experienced and recently established gender teams in the Netherlands. The interview guide and overview of decision moments are presented in Additional file 1. Interviews were audio-recorded and transcribed verbatim. Transcripts were analyzed using framework analysis [43]. After familiarization with the interviews, JS and at least one another researcher (AV, IH or BM) independently studied the transcript and highlighted quotes referring to decisional competence, the decision-making process, moral challenges, ways of dealing with these challenges, and needs for and ideas about receiving ethics support in this. Additional themes were also collected if deemed relevant to the overall study aim. The independent analyses were then compared and discussed to come at a coherent and as complete as possible overview. Subsequently, JS constructed an overview of all emerging themes including summaries and illustrative quotes from all interviews. After this, a first version of the tool was then drafted on the basis of this overview.

**Table 1:** Characteristics of 9 interviewed care providers

*Profession*
2 Child endocrinologists
2 Child and adolescent psychiatrists
4 Child psychologists
1 Nurse specialist
*Location*
4 From Center of Expertise on Gender Dysphoria (CEDG), Amsterdam UMC
2 From gender team Radboudumc Nijmegen
3 From periphery clinics providing parts of transgender care

### Phase 2: first draft for focus group meetings

The first version of the tool was discussed in two online focus group meetings for further refinement. For this, the interviewed care providers were invited. The aim and program of the meeting was to: (A) reflect on a presented overview of the interviews as a member check; (B) to collect eventual additional insights for this overview; (C) to apply the proposed content of the draft tool to actual cases, and (D) to collect feedback and guidance for further developing both content, structure and usability of the tool. The focus group meetings were facilitated by BM with AV and JS as assistants and observers, took two hours, were held online and audio-recorded. Summaries of these meetings was made by re-listening the audio-recordings. The experiences of the focus group meetings and the summaries formed the basis of an adapted, second version of the tool.

### Phase 3: pilot and feedback

A professional graphic designer turned the second version of the tool into a ready-to-use interactive pdf file which was pilot-tested for 3–5 weeks by four care providers. After testing the tool, they were interviewed about their experiences with the tool and they gave additional feedback. Furthermore, the tool was presented at team meetings and sent to the advisory board and other involved care providers to receive feedback.

### Phase 4: final tool presentation, implementation and evaluation

The final version of the tool was adjusted according to the collected feedback and subsequently sent to all care providers involved in the assessment and treatment of transgender and gender diverse adolescents, to use it in practice. Here, the care providers who were interviewed and involved in the previous phases were again involved by presenting the co-created tool themselves to their teams. They also took the lead in promoting and using the tool in practice. Six months later, an evaluation meeting was organized with the four care providers from the pilot phase to reflect on the current use and implementation, including potential barriers and ideas for further dissemination.

### Ethical considerations

Participation in the interviews and focus group meetings was on a voluntary basis. Interviewees and focus group members were informed about their possibilities to stop their participation at any stage with no necessity to give a reason. Prior to the start of each interview and the focus group meeting, written informed consent was obtained for conducting and audiotaping the session. The Institutional Review Board of AMC received and reviewed the research aims and methods, and declared that the study needed no further ethics approval according to Dutch regulations (Ref. no. W20_267).

## Results

### Phase 1: input from the interviews with care providers and earlier studies

In total, nine care providers were interviewed who had various professional backgrounds and worked at both centralized and periphery transgender care clinics, long existing as well as recently founded, in the Netherlands (see Table 1).

The interviews with care providers revealed five categories of needs and themes: (1) Need for guidance in assessing decisional competence and the decision-making processes with transgender adolescents; (2) Fertility, co-morbidity and role of parents as main moral challenges; (3) Current ways of dealing with moral challenges and (4) Preferred types of ethics support for the moral challenges. These categories were also found in the interviews with adolescents and their parents [42].

#### Need for guidance in assessing decisional competence and the decision-making processes with transgender adolescents

Care providers mentioned that they were not always sure how to assess the decision-making competence of young patients and that they would like to be better informed and educated (e.g., about measures for this). Some also questioned the existing four capacities (understanding, reasoning, weighing and making a choice) and clinical procedures for assessing decision-making competence and would like to receive more guidance here. For instance, one interviewee said “…that decision-making competence is a very vague concept. We do expect a certain extent of it but I think that we do not really know what we exactly want to see. To be able to repeat what puberty suppression is, I don’t know if I consider that as decision-making competence.” (R1).

Also, some respondents doubted the threshold to consider someone as sufficiently competent, especially regarding the long-term consequences, and expressed a need for consensus and guidance here as well. One asked for instance: “When do we consider a child as competent for decision-making? Because, […] to oversee the consequences, yes okay, but which consequences? The consequences for a year? For five years? Or for the future moment that they want a child? I feel a lot of struggle here. That I think, yes competent for the consequences in the upcoming period, but not when looking at the consequences for the really long term. But well, […] would you actually know those for any life decision? (R5). And according to another interviewee this was also hard regarding understanding potential side effects of treatment: “If you have never ever felt what a hot-flash is when you are twelve years old, which is completely normal, how could you say beforehand if you are able to manage that, if you have never experienced it?” (R9).

The uncertainty in (assessing) decision-making competence also seemed to be related to how interviewees identified and interpreted the puberty suppressing treatment. For some, this treatment was reversible, therefore, this treatment period could provide time to (further) investigating both the gender dysphoria as well as the decisional capacity, by more education and information about (long-term) possibilities and by actually starting to lively experience the preferred gender identity. According to one interviewee: “…I notice that the moment of decision-making is a bit expanded because, sometimes I feel like, puberty suppression can delay the process [of decision-making, red.] a bit as well. So that you can continue the process during that period, like, well, is this the step you really want to continue? […] Because, we are not yet in the hormonal treatment trajectory.” (R6). But others did consider puberty suppressing treatment as part of the hormonal transition to the other gender and, hence, as less reversible. These interviewees therefore put more emphasis on the importance to be well-informed about the long-term consequences and subsequent treatment options when making this first treatment decision on puberty suppression. For instance: “this [starting puberty suppressing treatment] really is an exciting decision because you can put children on a certain track where they might not easily get off, while you do not choose for permanent changes at the same time.” (R9). Some care providers explicitly struggled with the question whether or not puberty suppression could be considered as reversible treatment, as someone could be eligible to start with puberty suppressing treatment while it is already clear that this person might not be deemed competent for an irreversible treatment with stricter preconditions for informed consent, such as invasive surgery, for instance because of an intellectual disability that might hinder to become sufficiently informed and prepared for such an operation.

Interviewed care providers were explicitly asked about the moment at which they were consciously assessing the decision-making competence, by presenting a flowchart of all moments of decision (see Interview guide in Additional file 1). They all mentioned that their decision about decision-making competence was not bound to a particular moment but that they used many subsequent moments of meeting the adolescent to develop their impression of competence and that the final assessment was made together with their colleagues during the team meeting about the treatment indication. One said for instance: “…the idea that we look at it with multiple specialists gives me as care provider the feeling that I am not doing it [assessing decisional competence] on my own.” (R9). Care providers mentioned that, in general, they did not often doubt the decision-making competence of their minors and that they had not considered many clients as definitively incompetent for making decisions.

#### Fertility, co-morbidity and role of parents as main moral challenges

One topic was mentioned by almost all care providers: moral dilemmas about how to discuss the future child wish and current fertility preservation options with transgender adolescents. To cite one interviewee, “the thing that I feel the most with decision-making competence is: how can you actually know this at this moment? Because we know especially about fertility wishes that this can completely change, even separate from decision-making competence, it can even change between the ages of 30 and 35.” (R1). On the one hand, care providers emphasized the importance to inform the adolescent about the consequences of treatment for fertility, “…while on the other hand, I think that we are talking to a child who is not able at all to answer those questions” (R1). Especially the criterion for decision-making competence to oversee the consequences is under pressure here: "… so we are talking to children who still are in the Donald duck phase, about their fertility wishes. I think this is also difficult, as I am giving information about all possibilities, but I find it hard to state that, do they actually oversee the consequences?” (R8). The main reason for this difficulty is that the subject of discussion is exactly the thing which is in development: “…how could we let a child decide about something that he actually has not even half experienced yet?” (R6).

Also, co-existing psychiatric problems were perceived as potential barriers to involve the adolescent in the decision-making process and in assessing decision-making competence for the intended treatment. For instance, one interviewee said: “…with some very autistic patients, communication is always very complex. They can so desperately stick to certain things, things that they like, that they like a certain fabric of a dress for instance, so maybe they can oversee the image of what they want to become, but regarding all hormonal changes, it remains very hard to estimate if they can oversee those as well.” (R4).

Lastly, care providers were sometimes confronted with conflicting views between the transgender adolescent and their parents and had struggled with moral issues around the role of parents. In one of the interviews, the care provider mentioned to “sometimes have the feeling that parents see it from a different perspective, like that they can become a grandfather or grandmother and that they therefore stress the importance of preserving fertility, and for instance, […] a treatment like egg freezing, that they push on doing that. But this is so terribly invasive and intense, and traumatic for those biological girls to go into that trajectory when they feel to be a boy.” (R4). At the same time, moral issues around the role of parents could have many sides and nuances, as one interviewee explained: “Of course, these are the ethically difficult issues, in which you notice tensions between the wish of a child and the wish of parents. […] Child can be competent for decision-making but is maybe also competent enough to see like, well, if I do this it will clash so hard with my parents… maybe I should firstly put more energy in the systemic part so that parents can come along with us. But how long should you do that? How to act when parents keep on in refusing treatments?” (R8).

#### Current ways of dealing with moral challenges

The interviewed transgender care providers mentioned that, in general, they had good opportunities and an open and safe collegial climate to discuss their moral challenges with colleagues, in supervision or team meetings. In case of substantial doubts about the decision-making competence, the diagnostic trajectory prior to starting treatment was extended to invest in psycho-education, relationships and information in order to make children better capable to understand the treatment and consequences. For instance, one interviewee explained that they in a case of serious doubts, “then, in the end, we do discuss that with the youngster and parents: considering all that we know at this moment, […] we question if now is the right moment to start a treatment. Maybe you should develop a bit further so that things become more clear, for yourself as well, before entering a treatment phase” (R3).

#### Preferred types of support

To (better) deal with the moral challenges around the decision-making competence, the interviewees preferred to have a clear and shared overview of the definitions and assessment criteria for decision-making competence in this setting, “a card that presents different questions and various options, that would likely be helpful” (R6)”. At the same time, they stressed that they did not want a checklist or assessment test for competence, “I would definitely not want that, that you should reach like 80 percent [for competence] before being allowed to start, because that, for sure, is not the idea at all (R1)”.

Furthermore, they expressed the need for specific attention for how to approach vulnerable groups (e.g. those with psychiatric comorbidities) and a tool that enables a central and dynamic way to collect and store all information and ideas about the decision-making competence of the particular adolescent: “…like what do we know now, what do we have to take into account and at what age can we expect something, and at what moment not yet?” (R1). One interviewee added that it would be helpful to continue the tool over time: “So that you can observe, how is someone at the age of 9 and that you can do it again when someone is 13 years old, like has it been changed? That would be helpful for me. To see if a child is developing one selves.” (R5) Lastly, the tool should include guidance for the conversations with the adolescent and parents, and be focused on the moral aspects: “…I would like to have a tool that actually focuses on getting the underlying ethical dilemmas clearly on the table, which are the basis of my viewpoint (R8)”.

### First draft of the tool

The analysis of the interviews resulted in a first draft of the tool, constructed according to the five categories that had emerged from the interviews. This first draft consisted of four consecutive parts: clarifying information; identifying doubts and moral questions; guidance for conversations; and conclusions and next steps. Considering the questions and uncertainty regarding decision-making competence among care providers, it was important to provide clear information about decision-making competence in the tool. Furthermore, we wanted to provide explicit room for the mentioned moral challenges and make use of existing ways of dealing with them. Lastly, input about preferred types of support was taken into account when thinking about the use of the tool.

### Phase 2: feedback from the focus groups and further refinement

The draft tool was presented in two provider focus group sessions with respectively three and five participants (8 from the 9 interviewed care providers, one dropped out due to personal circumstances). They recognized the themes from the interviews and appreciated the 4-step structure of the tool. The dialogue about applying the tool to personal cases resulted in several questions to further clarify or add content to especially step 1 and 2. These steps were considered as very helpful but also complex. Focus group members found it hard to interpret the four criteria for decision-making competence in their context of care for transgender adolescents and missed consensus here. The criterion about appreciating the situation for instance: what does this mean when discussing treatment options with very young children? Do care providers apply this criterion in similar ways? Criteria might need more operationalization and concretization. Yet exactly this is subject of ongoing current debates, so this was deemed as unrealistic to achieve in this current process [24, 44]. Furthermore, some focus group members missed sufficient attention for how to act when adolescents are deemed decisional *in*competent. They also wondered if there were existing guidelines for fertility preservation in children or sharing decisions with persons with cognitive impairments. Completing the second step to identify doubts was also experienced as difficult. In addition, they desired more guidance in thinking about and formulating their values related to their moral doubts.

Furthermore, focus group members provided suggestions for the overall use and lay-out of the tool. They recommended the possibility for follow-up: to continue using the tool on different moments during the trajectory. They also wondered if a tool could be more tailored to the intended use, with different routes related to the aim, enabling users to skip certain steps or questions in the tool. In general, the ethics support tool was now perceived as too extensive with too many questions. Another recommendation was to include an exemplary case to inspire and help with completing the tool. Lastly, focus group members mentioned that the tool should also be feasible to complete as a team or with colleagues, as some found it rather one-sided if they completed it on their own.

The feedback from the focus group was processed and the following adjustments were made, which is indicated in the final tool in Table 2. In the first step, a starting question was added to help users define if their doubt involves the assessment of the decisional competence itself, or the way of dealing with a decisional (in)competent adolescent. Furthermore, extensive information was provided on decisional competence and decisional *in*competence, with reference to relevant guidelines. In the second step, the concept of moral doubt was explained and more questions were added to let users think about possibly related and sometimes conflicting values. The last step now provided explicit space to reflect on conclusions made in earlier steps to make an overall conclusion. Also, an exemplary case about moral challenges related to competency was presented as inspiration in every step.

**Table 2:** Final version of the ethics support tool: the competence consultant (in Dutch: de ‘Wilsbekwaamheidswijzer’)

A. Clarifying information	1. Clarify your starting point: What is your initial question?*
	I want to use this tool as I do not know what my doubt exactly is or because I (mainly) doubt about:
	• If this person is competent for the decision at stake (advice to do step A and eventually B, C and D)
	• How to involve this decisional *in*competent person in the decision-making process (advice to skip step A and do step B, C, D)
	• How to involve this decisional competent person in the decision-making process (advice to skip step A and do step B, C, D)
	2. Clarify the case:
	• Who is the client?
	• How sure are you about the gender dysphoria diagnosis, and is this relevant for your doubts?
	• What is the decision at stake?
	• Who is involved? Describe stakeholders shortly (parents/colleagues etc.)
	• What is the current situation?
	• What information do you miss at this point?
	3. Clarify the competence for the decision at stake:
	- Information box about decisional capacity (4 criteria, assessment, factors, incompetence, alternative views, external sources*)
	- Describe your impression for each of the 4 criteria
	- In case of incompetence: describe the representative persons*
	4. Clarify factors that might influence the competence
	- Factors that might play a role: age, psychiatric diseases, lack of supportive contexts and intelligence/intellectual disabilities
	- Describe which factors eventually play a role in this case
	- How to deal with these factors? (Examples: waiting or step-wise approach, investment in education and social system)
B. Identifying doubts and moral questions	1. Consider step A and think about: what do you find hard? What is your doubt?
	2. Does this refer to the treatment or diagnose, or to the competence and decision-making? Please mind that this tool is primarily meant for the latter
	3. What moral themes do you see? For example: fertility wishes, discussing consequences, vulnerable children, contact with parents*
	4. Clarify the values at stake and the perceived importance of these values
	- What values play a role? Examples in a word cloud: carefulness, happiness, beneficence, control, no harm, protection, respecting autonomy, best interests of adolescent, freedom, openness, attention, good care, solidarity, trust, information, respect, responsibility, tailoring care and togetherness*
	- Describe for each value: what do you have to do according to this value? What norm(s) is/are involved?*
	- Consider: what values are in conflict?*
	- Could you make a range of values from most to least important?
C. Guidance for conversations	1. Define the persons or parties that you need to talk with about your moral doubts or questions, and what you need to know from them
	2. Suggestions for conversations including helpful questions to identify their values at three levels: with colleagues, adolescent and parents:
	- Colleagues: options: 1-on-1 conversation, mono- or multidisciplinary team meeting, moral case deliberation. Discuss for instance: what is important for you as care provider(s)? It is OK if you think differently about this. In that case: describe the difference
	- Adolescent: reference to guidelines for shared decision-making, youth with intellectual disabilities and discussing fertility wishes. Discuss for instance: what is important for this adolescent? What values could you discover? Can you talk with this person about how he/she defines decisional competence? How does this add to your perspective?
	- Parents: potential topics to discuss: your own doubt, assess if they also feel doubtful, assess their perception on what is best for their child. Discuss for instance: What do parents find important? What values could you discover? What is their viewpoint on the decisional competence of their child? Can parents make the decision on behalf of their child?
D. Conclusions and next steps*	1. Make an overview here in order to make conclusions
	You can also use this page to guide the team conversation about your starting question*
	- What was your starting question? (See step A)
	- What are relevant and important values? (See step B)
	- How do involved stakeholders look at it? (See step C)
	2. What answer or conclusion could you give on the basis of the above? Describe here (eventually) the main considerations (as a team)*
	3. Reflect on your conclusions*
	- What are possible actions? What are (negative) consequences of these actions? For youth, parents, or yourself or the team?
	- How to deal with these negative consequences?
	4. What are the next steps?
	For example: make treatment plan with adolescent, enhance competence, re-assess competence, doing some parts of this tool again, inviting an ethicist or organizing moral case deliberation, etc
Extra information	Specific information about:
	- What is decision-making competence?
	- What are the four capacities of decision-making competence
	- Why should I assess decision-making competence?
	- How can I assess decision-making competence?
	- What factors influence decision-making competence of minors?
	- What to do in case of decision-making incompetence?
	- What are other viewpoints on decision-making competence?
	- Where can I go for more information?

*Adjusted or added element after feedback round in phase 2

### Phase 3: pilot phase

As mentioned, in phase 3 a professional graphic designer turned the second draft of the tool into a visually attractive and ready-to-use interactive digital document (pdf). Also, a name was given to the tool: “Competence Consultant” (In Dutch: “Wilsbekwaamheidswijzer”). The tool was distributed for a pilot test among four care providers who had been involved in previous focus group or interview phases. They completed the tool for at least one case individually, and one of them also used it to structure a team conversation. In the interviews about their experiences, they valued the user-friendliness: the clear structure and lay-out and having a central place for all relevant information. However, some mentioned that completing the tool was undesirably time-consuming. They therefore recommended to make the tool again shorter or easier to complete. For example, by navigating users by suggesting which steps can be skipped, depending on the intended use. They further experienced the second step rather difficult and wondered if an overview of exemplary values could be presented, for instance based on the interviews or related to the department itself. Another recommendation was to adjust the last step also into a guide for the team conversation.

The pilot tool version was furthermore sent to the other study participants from the interviews and focus groups and to the advisory board, for their written feedback. The pilot tool was finally also presented at a department meeting including an explicit call for feedback. This feedback was dominantly positive as many perceived the tool as highly relevant and welcome, but some warned that (eventual obligatory) use of the tool should not lead to extra administrative tasks or work pressure.

The feedback from both pilot users as well as written and oral impressions were processed in the third and final version of the tool, in collaboration with the graphic designer. Apart from some textual minor revisions, the main adjustments involved adding a text balloon at each of the starting questions to inform the user about which steps might be more or less useful for this question; a word cloud of possible relevant values in the second step and a revised title of the last step (‘Overview and Conclusions” instead of “Conclusions and next steps”). A screenshot of some pages of the final (Dutch) tool is presented in the Additional file 2 and an English overview of the steps and questions is given in Table 2.

### Phase 4: final tool presentation, implementation and evaluation

The final tool is an interactive infographic and includes four steps: (1) Clarify information; (2) Identify doubts and moral questions; (3) Guidance for conversations and (4) Overview and Conclusions. The tool can be used individually or as a group; notes can be made in the document itself.

The content of each step is described in detail in Table 2. In short, the tool starts with the step of clarifying and providing information to draw a clear and complete picture of the situation and intended use: why do you want to use the tool, who is the subject and what do you (need to) know about decision-making competence and potential relevant factors? The second step aims to identify and formulate the moral doubt and questions with helpful questions to indicate the source of the doubt, the moral theme and exemplary (potentially conflicting) values. In the third step, an overview is presented of suggestions for dialogues with others, focusing on clarifying and investigating their values. In the last step, an overview of the core elements from the previous steps can be made, followed by some questions to guide the team conversation towards a shared consideration and conclusion and plans for practice.

The final tool was distributed and its use was discussed with the gender team members via e-mail and during team meetings. The team suggested to use the tool by default when preparing, doing or reflecting upon the informed consent procedure and optionally in case of questions or doubts about the decision-making competence of a minor patient. In the subsequent months, the final tool was repeatedly presented during team meetings, yet care providers did not explicitly indicate to use the tool. In the evaluation session with the pilot users from the third phase, the researchers’ impression was confirmed that the tool had not yet frequently been used. The high workload, changes in the team, a lower number of diagnoses and treatments due to the corona crisis, and a lack of time to complete the tool were mentioned as main barriers, and the fact that the tool has not yet been integrated in existing administrative structures within the care system. At the same time, pilot users had received and experienced positive impact with using the tool themselves, especially in the sense that it helped them to clarify their doubts, formulate the dilemma and core question for the team meeting and to make concrete follow-up steps. To encourage further implementation, they suggested to start a new booster promotion campaign of the tool and to use the tool explicitly in upcoming planned moral case deliberation sessions with the teams, if the discussed case was related to decision-making competence.

## Discussion

This study described care providers’ moral challenges and perceived needs for ethics support around assessing decision-making competence in the care for transgender adolescents. The ethics support tool is specifically developed in and for the transgender care setting, even though it does make use of some general criteria for decision-making competence, since it involves unique moral challenges (such as the relationship between decision-making competence and the identity and existence of the transgender person). Based on a participatory research design, care providers and researchers co-created an ethics support tool for care providers. Firstly, the interviews revealed that care providers in this setting often struggled with the concept of decision-making competence, and that they were both looking for guidance in what decision-making competence entails and on how to assess it adequately. Secondly, the main moral challenges concerned (1) discussing (future) fertility wishes with the immature young children, (2) dealing with psychiatric problems and (3) the decision-making role of parents. Care providers sometimes extended the diagnostic process when they had serious moral doubts about the decision-making competence of their adolescent client. Lastly, care providers expressed a need for support in articulating their moral doubts, a structured way to discuss their moral doubts with their colleagues and practical suggestions for their conversations with the adolescents themselves and their parents. We will now reflect on our study by firstly considering the moral challenges and situate these into existing literature, followed by our reflections on co-creating an ethics support since this was a new and pioneering exercise. After this, we will zoom in on the tool itself, including limitations and strengths of the study, and we will end with a look forwards with implications for practice and research.

### Moral challenges around (assessment of) decision-making competence

The difficulty in assessing the capacities for decision-making competence is not new as it has been shown in earlier studies as well, also outside the transgender care setting [3, 13, 25]. It might partly be due to a lack of knowledge of existing measures to assess it (e.g., the MacCAT-T [45]). Competence could also inherently be considered as a morally complex concept since it always involves normative (hence, moral) questions, such as what is ‘good’ or ‘sufficient’ competence for decision-making by minors? [13]. The interviewed care providers also seemed to struggle with these moral questions, and some expressed a need for more consensus about this in their field. Besides, the decision-making competence is one of the major issues in the current debate on whether or not transgender and gender diverse adolescents should be deemed eligible to receive gender affirming treatment [8, 9, 15, 18, 19, 22]. The World Health Organization recently published a tool for healthcare professionals to assess and support adolescents’ decision-making competence, including a suggestion to use the MacCAT-t which further confirms the existing need for support in healthcare in general [46].

The interviewed transgender care providers in our study mentioned that, in general, they had not often assessed a transgender adolescent as (definitively) incompetent for decision-making. This is in line with an earlier study using a validated instrument to assess the decision-making competence of transgender adolescents [21]. Yet, this study further confirmed that, despite their competent clients, care providers still experienced various morally challenging issues related to decision-making competence in general, and related to the assessment of competence in particular, such as discussing long-term effects (e.g., for fertility), how to involve parents and dealing with psychiatric conditions (e.g., autism). These moral challenges were also mentioned by adolescents and parents themselves [16, 21]. Hence, the main moral struggle is not if these adolescents are considered competent but concerns the question on how to involve the adolescent who is still in development, despite being competent according to the four capacities, or despite being convincingly incompetent, in the decision-making process. This includes moral questions on how much an adolescent should understand the future consequences of both current and future treatment decisions, taking potential development of cognitive abilities and thus an expected increased understanding into account; how to deal with an expected lack of this development in case of intellectual disabilities or impeding psychiatric conditions; and when the adolescent’s wishes deserve priority over those of parents, if these are in conflict (or vice versa: when can a parent adequately provide informed consent for their transgender offspring in the case that they are not (yet) deemed competent). In the end, the focus should lie on supporting the adolescent’s right for making and expressing a decision by themselves, as was recently also pleaded for by Ashley [44]: ‘because, regardless of patients’ competence, there is typically nobody who is better positioned to make medical decisions that go to the heart of the patient’s identity than the patient themselves’ and therefore, ‘emphasis should be put on supporting, rather than allocating, decision-making around gender-affirming care.’

### Co-creating an ethics support tool

Based on the input from the care providers, an ethics support tool was developed in the second phase of our study. The tool is focused on and tailored to the actual and complex practice of transgender adolescent care and is thereby an innovative type of ethics support as it also provides thematic guidance, which is not the focus of other forms of ethics support, such as moral case deliberation. This process of co-creation resembles our approach in earlier studies in which ethics support tools were developed [33, 35, 36, 47]. It can be characterized as an ‘integrative ethics support’ approach because both the process as well as the content dissemination took place within the actual care practice of transgender care. Care providers themselves were made responsible and, hence, co-owner of the process and product. The final tool can be seen and used as an example of the so-called ‘innovative [Clinical Ethics Support] activities through an emerging design’ [36]. Here, the ethicist acts as a facilitator of the dialogical process instead of a (sole) creator of the content, as Inguaggiatto et al. [48] have stressed that ‘ethical knowledge develops through an exchange of perspectives on a specific situation by those who experience it as morally troublesome’. As ethicists, we however faced moral challenges during this exercise ourselves, in a way comparable to challenges of care providers in the decision-making process with their clients, such as: how to balance being supportive and being directive at the same time during the ethics support process? When, and if so, to what extent, should we as ethicists take a more leading role in defining and structuring the content? Furthermore, when reflecting on the process, we might have missed the input from adolescents themselves. Although our study was focused on the challenges of care providers, it might have enriched our process—and potentially also the product—when adolescents were also involved in one or more phases. Finally, it was not always easy to interpret the moral issues raised by care providers as being related to either decision-making competence at the one hand or treatment decisions and/or gender dysphoria at the other hand. The latter was not our main interest while we realize that they are closely related to each other.

### The Competence Consultant

The final tool, the Competence Consultant, integrates both the ‘know-how’ (e.g., information and guidelines) as well as the moral doubts regarding decision-making competence. It does so by providing a stepwise framework for firstly considering and collecting relevant information, after which the moral doubt(s) can be defined and deepened. Subsequently, the values and voices of the adolescent, parents and colleagues can be investigated in order to come to a well-considered overview and outlook to next steps in the decision-making process. These steps can be performed either with the team or individually as care provider. The tool hereby stimulates care providers themselves to become more morally sensitive and competent in recognizing and formulating moral challenges, and offers ways to discuss these challenges as well. It is important to stress that the tool in itself does not aim to, and therefore does not take any stance in, assessing transgender adolescents as definitively competent or incompetent for decision-making. Yet, we did observe and learn from care providers that they most often do not doubt the competence [21].The assessment of decision-making competence cannot be solely done with a checklist as it remains a dynamic and intangible concept [13]. As such, the tool provides care providers with guidance to both formulate the moral doubt or question as well as to find the answer, although the tool does not give the answer (hence, no directiveness) but by stepwise structuring the consideration (explicitly emphasizing the need for dialogue with adolescent and colleagues) and referring to existing supportive instruments. We do either not (aim to) solve or answer the current controversies in transgender youth care, which were described in the introduction. Yet, we do contribute to the complex debate by a helpful and practice-oriented framework for the care providers to unravel moral challenges, articulate uncertainties and make well-considered conclusions on a case-by-case basis, thereby preventing potential misunderstandings about decision-making capacity of minor’s and stimulating careful assessments in practice. Especially since the field of transgender care still lacks evidence-based consensus regarding treatment options, shown in recent critics on existing medical approaches [49], our ethics support tool is highly needed and relevant for the individual care provider.

### Limitations

Our approach to co-creating an ethics support tool also had some shortcomings. Firstly, the co-created tool might have a limited scope as it is tailored to the needs and use of care providers, with no explicit step or task for clients and/or their parents. This is not surprising since the project did explicitly aim to develop a tool for care providers. While we did include the input from adolescents and parents indirectly via the results of other studies, it could be seen as a weakness that adolescents and parents did not have a larger role in this process since it is important to involve them as well and give them a fair say in this process. Another limitation might be that the pilot phase only involved four care providers, due to time constraints. We however also collected feedback during this phase from as many as possible care providers in general team meetings. Lastly, the actual use of the tool by care providers was limited in the first period after its launch, due to the hectic period of the corona measures, high workload and societal debates in which the gender team had to work during our study period. Despite some first positive experiences, the tool clearly needs further implementation and feasibility evaluation.

### Strengths

Given the centrality of decision-making competence in adolescent transgender care and the ethical challenges and critical debate around early medical-affirming interventions, the main strength of our study is that we developed an actual and user-friendly hands-on tool to use in clinical practice, based on the needs of care providers and stakeholders, with positive evaluations of care providers who were helped in dealing with moral issues around the decision-making competence of adolescents in transgender care. In comparison with two existing tools, the WHO tool [46] and the MacArthur Competence Assessment Tool [45], this ethics support tool not only focuses on specific contextual *moral* challenges of the professionals in transgender care but it also supports the professional in clarifying *their* specific (moral) challenges. In addition, the design of this ethics support tool allows for easy use, virtual storage and the ability to use it over time, which makes it simply applicable. The final tool contains a broad overview of relevant information concerning decision-making competence in this field, helpful questions to formulate the moral doubt and practical suggestions for the dialogue with adolescent, parents and colleagues and references to existing guidelines. The developmental process can be seen as a case example for how to apply general theories (like the criteria for decision-making competence) to a practical tool in a specific setting.

### Implications for practice and research

The tool can help healthcare professionals when dealing with morally challenging situations around the decision-making processes of transgender and gender diverse adolescents. It does not solve the dilemmas however but helps to further clarify and investigate these issues. It can serve as an addition to a careful clinical approach in cases where moral concerns exist and may of help to ensure that transgender care is provided to those adolescents who need it in a conscientious way. Insight into the usage of the tool can also inform transgender care providers about repeatedly occurring moral questions, in order to develop (normative) policies and clear guidance on how to deal with and answer these questions. In future, the actual use of the tool should be further encouraged and integrated within existing working structures.

The tool can also be translated and adapted for international use in the transgender care setting. In this setting, the tool can also be used as a starting point for developing new frameworks and guidelines for shared decision-making, including attention for diagnosing and defining gender dysphoria itself and adequately involving all stakeholders. Furthermore, in an adapted form, the tool might also be helpful in other settings in which moral dilemmas occur about the decision-making competence of adolescent clients. Since co-creating a tool for ethics support in particular settings is a promising yet pioneering form of clinical ethics support, it will also require further conceptualization and task descriptions of both process (co-creation) as well as the product (the content of the tool itself).

## Conclusion

As many moral dilemmas exist in the care setting of transgender and gender diverse adolescents, this study provided an overview of these moral dilemmas and developed a practical ethics support tool, the so-called ‘Competence Consultant’, to help transgender care providers to formulate, consider, discuss and deal with these moral dilemmas, thereby contributing to their moral sensitivity. The tool and the developmental process contribute both to the field of innovative ways of offering ethics support on how to establish a process and product of co-creation between ethics support staff and care providers. It thereby offers support for the field of emerging and ongoing moral issues concerning the decision-making process with adolescents in all phases and practices of transgender care.

## Supplementary Information

**Additional file 1.** Interview guide and overview of decision-moments.

**Additional file 2.** Screenshots of the first pages of the Ethics Support Tool, ‘deWilsbekwaamheidswijzer’.

## Data Availability

The datasets used and/or analysed during the current study are available from the corresponding author on reasonable request.

## Abbreviations

CEDG: Center of Expertise on Gender Dysphoria
MacCAT-T: MacArthur Competence Assessment Tool for Treatment
