# Supplementary material for: How to deal with moral challenges around the decision-making competence in transgender adolescent care? Development of an ethics support tool
Source: BMC Med Ethics. 2022 Sep 22;23:96. doi: 10.1186/s12910-022-00837-1 (PMC9494804; doi:10.1186/s12910-022-00837-1)

Appendix I Interview guide and overview of decision-moments

Interview guide

General introduction

1. What role do you have in the treatment of transgender adolescents?
2. In which decisions are you involved?

Current experiences with decision-making processes and decision-making competence

3. Can you describe how you arrive at decisions concerning starting puberty suppression with children/youth with gender dysphoria?
4. What aspects do you consider in this decision-making process?
5. How important is it according to you to assess the decision-making competence?
6. How do you assess the decision-making competence? Implictly? Do you use an instrument like the MacCAT?
7. What do you further take into account here? Age? IQ? Life experience?
8. What does it mean for the further trajectory if the child is obviously competent for decision-making?
9. What do you in case of doubt? Do you have an example of this?
    a. What makes you doubt?
    b. Did this influence the further trajectory with this child? If so, how?
10. What do you if you are convinced that a child is *not* competent for decision-making? Do you have an example of this?
11. What role do parents have in the decision-making process? And around assessing decision-making competence?
12. The moral dilemmas in transgender care have earlier been studied. Do you agree with the finding that many moral dilemmas arise in this setting? What do you find most difficult?
13. How do you deal with these challenges? What do you do when you experience a dilemma?

Ideas about and needs for a tool

14. Do you want more support in dealing with those moral challenges? Why (not)?
    a. If so, could you indicate where you want to be supported (mostly) during the trajectory? See the overview on the next page.
    b. If not, could you imagine that others might need support here? Where during the trajectory?
15. What should be included in a certain tool for support? Think of the earlier described moral challenges. Guidance in assessing competence? Referrals to existing guidelines? Stepwise framework for informed consent procedures? General tips for the decision-making process?
16. What should the tool be focused on, specifically? Think of moral dilemmas, doubts, decision-making competence, information, etc.
17. What would you like regarding lay-out, structure or format of the tool? Think of a map, flyer, poster, conversation method, etc.
18. What do you expect if you are going to use a tool?
19. How should we evaluate the tool? What should be improved by using it? What endpoints do you see?
20. What should we further take into account when developing a tool?

Overview of decision moments care provider and transgender adolescent

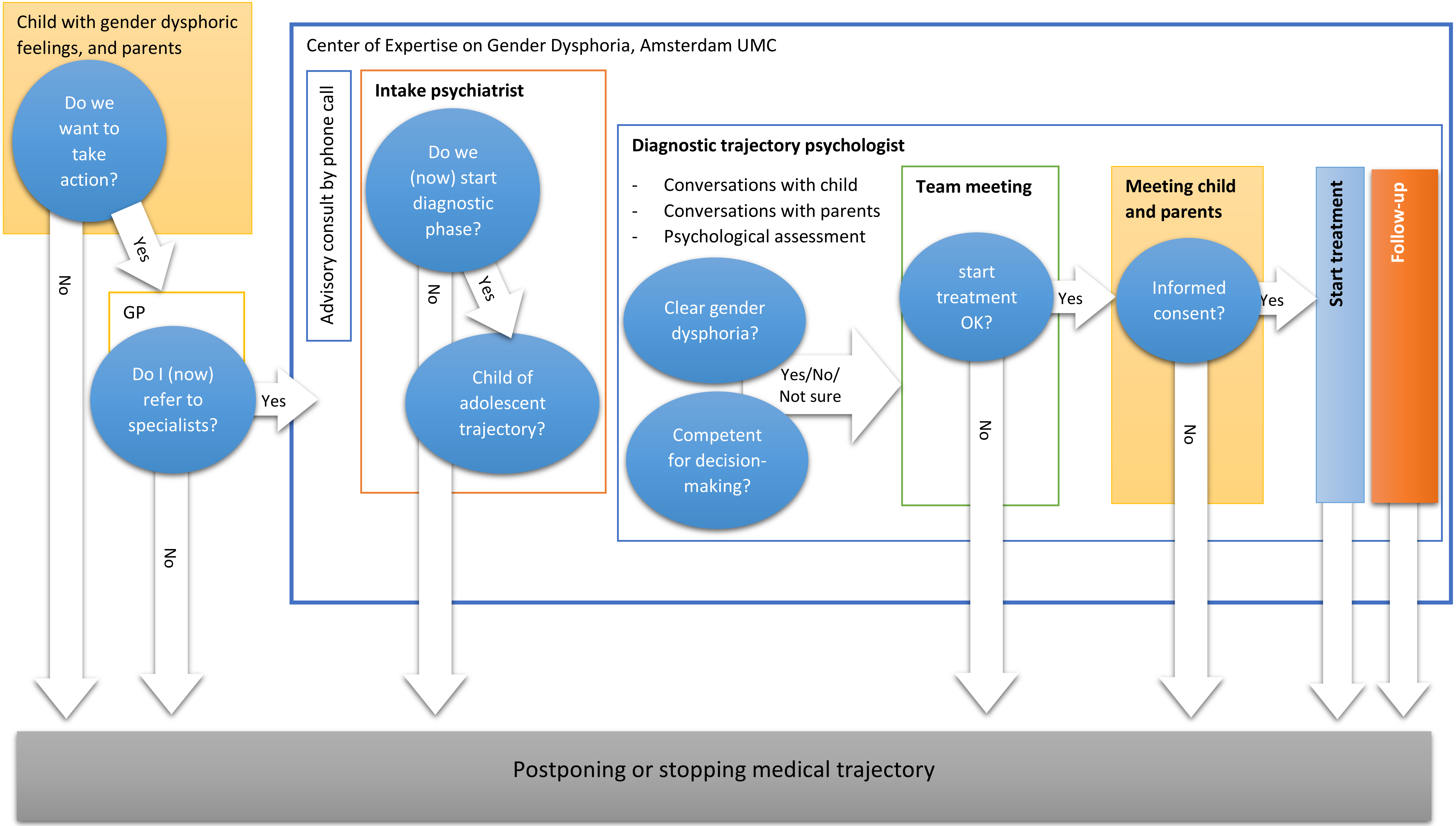

Supplement: Supplementary file 1 — Additional file 1. Interview guide and overview of decision-moments. [file 12910_2022_837_MOESM1_ESM.pdf]
