# Supplementary material for: How to deal with moral challenges around the decision-making competence in transgender adolescent care? Development of an ethics support tool
Source: BMC Med Ethics. 2022 Sep 22;23:96. doi: 10.1186/s12910-022-00837-1 (PMC9494804; doi:10.1186/s12910-022-00837-1)

Appendix II **Screenshots of the first pages of the Ethics Support Tool, 'de Wilsbekwaamheidswijzer'**

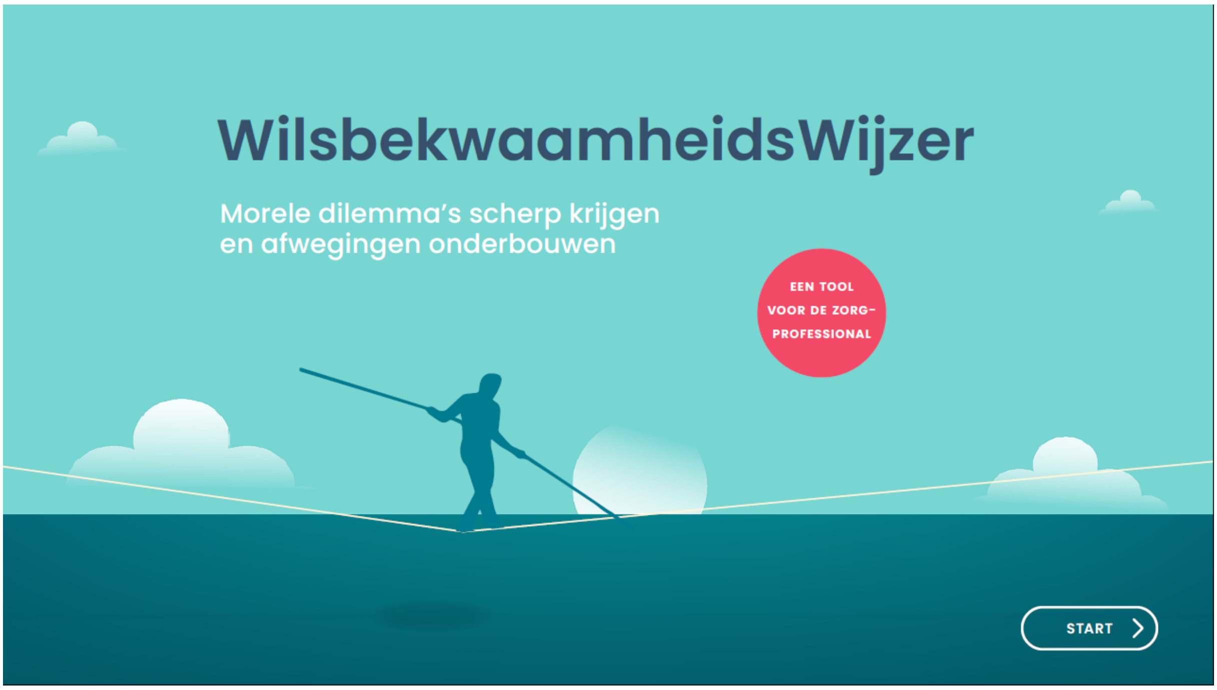

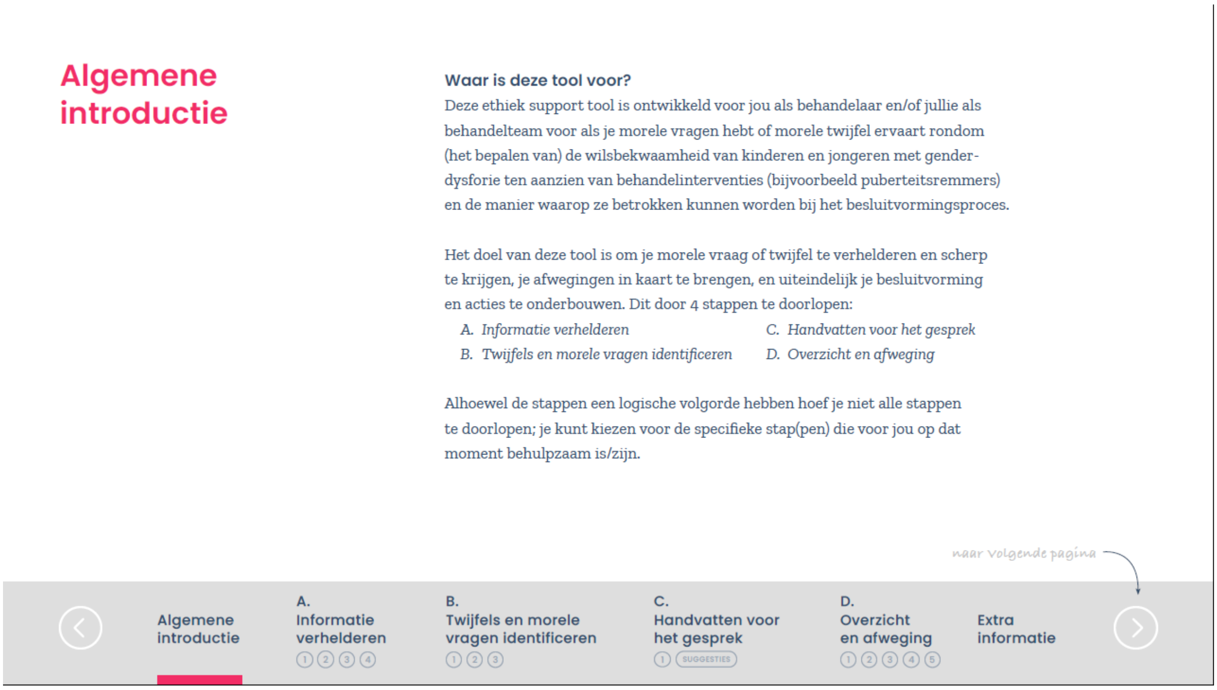

Algemene introductie

Waar is deze tool voor?

Deze ethiek support tool is ontwikkeld voor jou als behandelaar en/of jullie als behandelteam voor als je morele vragen hebt of morele twijfel ervaart rondom (het bepalen van) de wilsbekwaamheid van kinderen en jongeren met gender-dysforie ten aanzien van behandelinterventies (bijvoorbeeld puberteitsremmers) en de manier waarop ze betrokken kunnen worden bij het besluitvormingsproces.

Het doel van deze tool is om je morele vraag of twijfel te verhelderen en scherp te krijgen, je afwegingen in kaart te brengen, en uiteindelijk je besluitvorming en acties te onderbouwen. Dit door 4 stappen te doorlopen:

A. *Informatie verhelderen*
B. *Twijfels en morele vragen identificeren*
C. *Handvatten voor het gesprek*
D. *Overzicht en afweging*

Alhoewel de stappen een logische volgorde hebben hoef je niet alle stappen te doorlopen; je kunt kiezen voor de specifieke stap(pen) die voor jou op dat moment behulpzaam is/zijn.

naar volgende pagina

Algemene introductie | A. Informatie verhelderen ① ② ③ ④ | B. Twijfels en morele vragen identificeren ① ② ③ | C. Handvatten voor het gesprek ① SUGGESTIES | D. Overzicht en afweging ① ② ③ ④ ⑤ | Extra informatie

Stap A.
Informatie verhelderen

1. **Verhelder je startpunt: wat is je uitgangsvraag?**
Ik pak deze tool omdat ik niet weet waar mijn twijfel precies zit of omdat ik (met name) twijfel heb rondom de vraag:
(vink aan)
- ☐ Of deze persoon wilsbekwaam is ten aanzien van de beoogde behandelbeslissing. ⓘ
- ☐ Hoe ik deze wilsonbekwame persoon kan betrekken bij het besluitvormingsproces. ⓘ
- ☐ Hoe ik deze wilsbekwame persoon kan betrekken bij het besluitvormingsproces. ⓘ

Klik hier

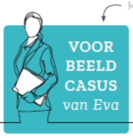

VOORBEELDCASUS

In deze WilsbekwaamheidsWijzer hebben we een voorbeeldcasus opgenomen zodat je kunt zien hoe het daar is ingevuld.

Klik op het icoon op de pagina om de voorbeeldcasus te openen.

Stap D.
Overzicht en afweging

1. **Maak hieronder een kort overzicht om een afweging te maken.**
Deze pagina kun je ook gebruiken als handvat voor het teamoverleg/MDO over je uitgangsvraag.

a. Wat was je/jullie uitgangsvraag? (*klik **hier** voor stap A*)
Beschrijf kort hoe je/jullie als team er vooraf aan het overleg in staat/staan.

b. Welke waarde(n) is/zijn hier voor jou/jullie relevant, en welke is/zijn het belangrijkst voor het omgaan met de morele twijfel? (*zie eventueel **hier** voor stap B*)

c. Hoe kijken de betrokken partijen (*jeugdige/ouders/collega's etc.*) naar deze vraag? (*zie eventueel **hier** voor stap C*)
Indien het om een wilsonbekwaam persoon gaat: neem ook de visie van de vertegenwoordigers mee!

Klik hier

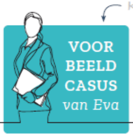

Supplement: Supplementary file 2 — Additional file 2. Screenshots of the first pages of the Ethics Support Tool, ‘deWilsbekwaamheidswijzer’. [file 12910_2022_837_MOESM2_ESM.pdf]
